# Reduced immune function predicts disease susceptibility in frogs infected with a deadly fungal pathogen

**DOI:** 10.1093/conphys/cow011

**Published:** 2016-04-15

**Authors:** Anna E Savage, Kimberly A Terrell, Brian Gratwicke, Nichole M Mattheus, Lauren Augustine, Robert C Fleischer

**Affiliations:** af1 Center for Conservation and Evolutionary Genetics; af2 Center for Species Survival and; af3 Center for Animal Care Sciences, Smithsonian Conservation Biology Institute, National Zoological Park, 3001 Connecticut Avenue NW, Washington, DC 20008, USA

**Keywords:** Amphibian, bacterial killing ability, *Batrachochytrium dendrobatidis*, chytridiomycosis, immune response, *Lithobates yavapaiensis*

## Abstract

The relationship between amphibian immune function and disease susceptibility is of primary concern given current worldwide declines linked to the pathogenic fungus *Batrachochytrium dendrobatidis* (*Bd*). We experimentally infected lowland leopard frogs (*Lithobates yavapaiensis*) with *Bd* to test the hypothesis that infection causes physiological stress and stimulates humoral and cell-mediated immune function in the blood. We measured body mass, the ratio of circulating neutrophils to lymphocytes (a known indicator of physiological stress) and plasma bacterial killing ability (BKA; a measure of innate immune function). In early exposure (1–15 days post-infection), stress was elevated in *Bd*-positive vs. *Bd*-negative frogs, whereas other metrics were similar between the groups. At later stages (29–55 days post-infection), stress was increased in *Bd*-positive frogs with signs of chytridiomycosis compared with both *Bd*-positive frogs without disease signs and uninfected control frogs, which were similar to each other. Infection decreased growth during the same period, demonstrating that sustained resistance to *Bd* is energetically costly. Importantly, BKA was lower in *Bd*-positive frogs with disease than in those without signs of chytridiomycosis. However, neither group differed from *Bd*-negative control frogs. The low BKA values in dying frogs compared with infected individuals without disease signs suggests that complement activity might signify different immunogenetic backgrounds or gene-by-environment interactions between the host, *Bd* and abiotic factors. We conclude that protein complement activity might be a useful predictor of *Bd* susceptibility and might help to explain differential disease outcomes in natural amphibian populations.

## Teaser

Amphibians are susceptible to a disease-causing fungus known as Bd. We experimentally infected lowland leopard frogs to determine if Bd infection is associated with altered immune function in the blood. Our study suggests that Bd infected frogs with less active defense proteins in the blood are more susceptible to this deadly fungus.

## Introduction

The pathogenic fungus *Batrachochytrium dendrobatidis* (*Bd*) was first identified as the causative agent of chytridiomycosis and definitively linked to declines and extinctions of natural amphibian populations in 1998 (Berger *et al*., 1998). Five years earlier, immunosuppression had been suggested as a potential explanation for enigmatic amphibian declines (Carey, 1993). Yet only recently has it been demonstrated that host immune function plays a role in *Bd* susceptibility (Ribas *et al*., 2009; Savage and Zamudio, 2011; Rosenblum *et al*., 2012) and that the pathogen actively suppresses host immunity by inducing lymphocyte apoptosis (Fites *et al*., 2013). These findings highlight the need to gain a better understanding of amphibian immune responses, particularly in the context of *Bd* infections.

The genomic basis of immunity is highly conserved across vertebrates (Flajnik and Kasahara, 2001; Nonaka and Kimura, 2006; Ohta *et al*., 2006). Many of the major structural and functional elements of innate and acquired immunity in mammals are also present in amphibians (Robert and Ohta, 2009; Zimmerman *et al*., 2010a,b), although certain aspects of this system, such as lymph transport, are unique to anurans (Hedrick *et al*., 2013). Vertebrates share a complex network of serum proteins, known as the complement system, that plays a key role in both innate (Koppenheffer, 1987) and acquired immunity (Nielsen and Leslie, 2002). Through a co-ordinated cascade of reactions, these proteins trigger local inflammatory responses, promote the uptake and destruction of pathogens by phagocytes, and form membrane-attack complexes that destroy certain pathogens directly (Janeway *et al*., 2001).

Innate immunity in general, and serum complement in particular, appears to be more diverse and functionally more important for immunity in ectothermic compared with endothermic vertebrates (Sunyer *et al*., 1998; Zhu *et al*., 2005). Crocodilian serum complement activity has a broader spectrum of bacterial killing ability (BKA) compared with human serum (Merchant *et al*., 2003), as also in teleost fish, which express at least five isoforms of the C3 serum complement protein component (Sunyer *et al*., 1997). In the frog *Bombina maxima*, whole-transcriptome profiling resulted in the identification of 157 highly expressed complement transcripts, including C2, C3, C3d, C4a, C5, C6, factor B, factor D and factor I (Zhao *et al*., 2014), confirming that anuran complement proteins also show expanded diversity compared with endotherms. Functional studies reveal a more complex pattern; complement gene expression in catfish infected with two bacterial species was up-regulated in response to *Edwardsiella ictaluri* but down-regulated in response to *Flavobacterium columnare* (Jiang *et al*., 2015), whereas these genes were down-regulated among *Rana muscosa* and *Rana sierrae* infected with *Bd* (Rosenblum *et al*., 2012). Thus, complement activity is an important immune defense for ectothermic vertebrates, but its role in combating infection is clearly pathogen and host specific.

Whether the complement system of amphibians is activated in response to *Bd* infection is unclear. Complement proteins are present in human epidermis (Dovezenski *et al*., 1992), and complement activation plays a key role in inflammatory responses to certain epidermal fungal infections in mice (Ray and Wuepper, 1978) and humans (Tagami, 1992). Amphibians with chytridiomycosis typically demonstrate minimal skin inflammation (Pessier *et al*., 1999; Berger *et al*., 2000, 2005; Densmore and Green, 2007), suggesting that *Bd* does not trigger a sufficient immune response to activate complement pathways. However, immunological responses to *Bd* infection vary among amphibian species, and dermal inflammation was observed in up to 40% of *Litoria caerulea* with chytridiomycosis (Berger *et al*., 2005). Furthermore, experimental infection of *Rana cascadae* with *Bd* increased BKA, a measure of combined cellular and humoral immune function (including complement activity; (Gervasi *et al*., 2013). Yet the opposite effect was observed in *Pseudacris regilla*, where *Bd* infection decreased blood BKA (Gervasi *et al*., 2013). In contrast to whole blood, analysis of plasma or serum bactericidal ability can provide more specific information about complement activity, because these fluids contain only humoral components of the immune system. Plasma bactericidal assays have been used to identify significant associations between environmental factors and innate immune function in snakes (Sparkmann and Palacio, 2009), alligators (Merchant *et al*., 2003), turtles (Zimmerman *et al*., 2010a,b), fish (Hayman *et al*., 1992), salamanders (Terrell *et al*., 2013) and frogs (Maniero and Carey, 1997; Venesky *et al*., 2012).

Cellular mediators of immunity (e.g. lymphocytes) are also present in the epidermal layer and are known to be influenced by *Bd* infection (Fites *et al*., 2013). In addition to inhibiting lymphocyte function, *Bd* may alter the distribution/production of amphibian leucocytes, although this topic is largely unexplored. Percentages of circulating neutrophils and eosinophils were significantly reduced in *Bd*-infected tree frogs (*Litoria chloris*) compared with uninfected individuals (Woodhams *et al*., 2007). Circulating leucocytes represent a primary line of defense against invading pathogens (Jain, 1993). Numbers and proportions of leucocytes in peripheral blood are commonly used indicators of infection and physiological stress in amphibians and other vertebrates (Davis *et al*., 2008; Campbell, 2015). Neutrophils and lymphocytes constitute the majority of leucocytes in amphibian peripheral blood (Bennett *et al*., 1972; Cathers *et al*., 1997). Across vertebrates, the relative proportion of these two cell types (the N/L ratio) changes predictably in response to stress (Bennett and Alspaugh, 1964; Bennett *et al*., 1972; Turner, 1988) or infection (Davis *et al*., 2010; Gervasi *et al*., 2013). Specifically, proportions of neutrophils increase, whereas proportions of lymphocytes decrease, reflecting changes in leucocyte production/distribution (Davis *et al*., 2008). Differentiating the causes of increased N/L ratios can be challenging, particularly given that stress and disease are closely related (Blaustein *et al*., 2012). Nonetheless, leucocyte profiles provide valuable information on immune physiology, particularly when only small blood volumes are available for analysis. Additionally, these cells respond to stressors more slowly than glucocorticoids, reducing the likelihood that animal handling will confound stress measurements based on leucocyte profiles compared with hormone assays (Davis *et al.*, 2008).

The lowland leopard frog (*Lithobates yavapaiensis*) is a good candidate model species for investigating the determinants of chytridiomycosis susceptibility. Natural populations have persisted in the southwestern USA with *Bd* infection and associated mortality for at least 25 years (Bradley *et al*., 2002; Sredl and Jennings, 2005). Mortality is ongoing in some populations but not others (Savage *et al*., 2011), a pattern that is predicted by population genetic variability (Savage *et al*., 2015). Host immunogenetic variation among *L. yavapaiensis* individuals is associated with survival in experimental *Bd* infections (Savage and Zamudio, 2011), implicating variation in immune function as a predictor of *Bd* infection outcomes. However, functional immunity has not been measured in *L. yavapaiensis*. Thus, immunogenetic variation might be indirectly linked to disease through a correlation with total genetic variation rather than a direct, functional relationship. An exploration of immune physiology in *L. yavapaiensis* is an important step in resolving functional relationships among disease, genetics and immunity.

Here, we experimentally exposed *L. yavapaiensis* to *Bd* in order to identify changes in humoral and cellular measures of immune function during *Bd* infection. We measured survival, zoospore load, body mass, plasma BKA and circulating leucocyte profiles to test the following hypotheses: (i) immune responses to *Bd* infection are energetically costly and include elevated complement activity and changes in leucocyte distribution/abundance; (ii) frogs that survive infection are able to mount an effective immune response and demonstrate increased complement activity, greater body mass, elevated N/L ratios and lower infection intensities compared with frogs dying of chytridiomycosis.

## Materials and methods

### Animal husbandry

We collected *L. yavapaiensis* individuals from the Muleshoe Ranch Cooperative Management Area in Graham County, AZ, USA. This population has high chytridiomycosis susceptibility in controlled infections (Savage and Zamudio, 2011) but low morbidity and mortality in the wild owing to a geothermal spring that limits chytridiomycosis (Schlaepfer *et al*., 2007; Forrest and Schlaepfer, 2011; Savage *et al*., 2015). We collected partial egg masses (50–75 eggs per clutch) on 15 September 2012 and shipped them overnight to the National Zoological Park (NZP) in Washington, DC, USA. Clutches 1 and 2 were collected from the Secret Spring pond [Universal Transverse Mercators (UTMs): 571093E, 3578368N], clutches 3–5 were collected from the upper thermal spring pools (UTMs: 571502E, 3570135N), and clutches 6 and 7 were collected from the main thermal spring pool (UTMs: 571681E, 3578002N). Upon hatching, larvae spent 30 days in quarantine and were confirmed by NZP veterinary staff and Genetics Laboratory staff to be in good health and free of *Bd* and ranavirus by PCR testing. We then reared individuals through metamorphosis in an animal holding room at the NZP’s Reptile Discovery Center at 24 ± 3°C with a 12 h–12 h light–dark cycle and 5% UVB during lighted hours. Animals were reared in plastic mouse containers filled with reconstituted water (reverse osmosis water with salts added) in groups of 10–15 individuals. We rotated clutch mates among cages to equalize numbers per cage owing to moderate levels of tadpole mortality (∼20% of tadpoles died over 7 months). Cage locations were rotated bi-weekly to avoid any potential micro-environmental effects. Tadpoles were fed a diet of 90% dried spirulina algae and 10% fish food flakes, egg whites and bee pollen *ad libitum*.

When all frogs had reached or exceeded Gosner stage 41, we transferred them to a climate chamber held at 20 ± 2°C with a 12 h–12 h light–dark cycle and 5% UVB during lighted hours. Metamorphosed juvenile frogs were housed individually in plastic mouse cages held at a slight angle, with a reservoir of water at one end and a plastic shelter with wet paper towels at the other end. Frogs were fed vitamin-dusted crickets, mealworms and wingless flies daily *ad libitum*.

### Experimental *B. dendrobatidis* infections

On 4 April 2013 (2–4 weeks post-metamorphosis for all individuals, weight range 1.1–2.9 g), we placed all frogs in individual plastic infection chambers (sterile plastic containers 12 cm in diameter, with holes poked in the lids) with a film of water at the bottom and either ∼100 000 zoospores of *Bd* in 1 ml of reconstituted water (*n* = 49 *Bd*-exposed frogs) or 1 ml of reconstituted water only (*n* = 28 sham-exposed frogs). We exposed frogs to *Bd* strain JEL423, isolated from an *Agalychnis lemur* individual in Panama in 2004 and cryopreserved until 7 days before infections. After 24 h in the infection chambers, we returned all frogs to their plastic mouse cages and resumed husbandry as described above. Gloves were worn while handling frogs and, subsequent to infection, were changed between cages to avoid pathogen spread.

Every 7 days, starting on day 1 post-infection, we swabbed the epidermis of each individual using sterile fine-tip swabs (Medical Wire and Equipment Co. MW113) following standardized protocols (Hyatt *et al*., 2007) and stored swabs dry in 2 ml tubes at −80°C. Frogs were euthanized following a staggered schedule to allow comparison of immune function during early (≤day 15) vs. late infection (≥day 29). Given the small body mass of each frog, immune function could be assessed only once in each individual, after euthanasia. On days 1, 4, 8, 15, 29 and 41 post-infection, eight frogs (five *Bd* exposed and three control) were swabbed and then euthanized. We selected these individuals semi-randomly; using a random number generator, we chose *Bd*-exposed and control individuals separately for euthaniasia, eliminating the entire clutch from the selection pool after one individual from that clutch was selected. Thus, we ensured that euthanized frogs represented the broadest genetic sampling possible, with paired *Bd*-exposed and control frogs from three clutches per euthanasia day. On day 55 after *Bd* exposure, we ended the experiment and euthanized all surviving frogs. At any time point during the experiment, an individual manifesting all signs of chytridiomycosis (ventral redness, skin shedding, lethargy, anorexia and loss of righting ability) was euthanized by an NZP veterinarian to prevent further suffering and ensure that fresh blood was collected before death. Immediately before euthanasia, we swabbed each frog for *Bd* quantification. We then injected each frog with ∼200 μl of MS-222 (10 g/l in sterile water buffered to a pH of 7 with sodium bicarbonate). Each frog was injected into the body wall on the right flank using a sterile 1 ml syringe and a 25 gauge ¾ inch needle. A new pair of sterile gloves was used to handle each frog.

### Blood collection

We placed each euthanized frog on an ethanol-sterilized surface covered with fresh kimwipes and used sterilized scissors, tweezers and scalpels to open the body cavity and sever the aorta. We then used sterile heparinized capillary tubes to collect as much blood as possible (one to three tubes depending on the size of the animal). A new pair of sterile gloves was used to handle and collect blood from each frog. A small volume (5 µl) of blood was immediately smeared onto a glass microscope slide for differential leucocyte counts. The remainder of each blood sample was stored on ice and centrifuged (5 min, 2000***g***) within 2 h to isolate plasma for bacterial killing assays. Blood plasma was transferred to a 0.5 ml plastic tube and stored at −80°C until bacterial killing assays were performed. Corticosteroids are a more traditional indicator of stress (Davis *et al.*, 2008), but we did not have sufficient blood from these small metamorphs to include both a corticosterone analysis and the BKA analysis; therefore, we prioritized the BKA analysis as an indicator more directly linked to immune function.

### Quantification of *B. dendrobatidis*

We extracted DNA from swabs using Qiagen DNeasy blood and tissue kits, eluting into a volume of 200 µl for all samples. The number of *Bd* genome equivalents (GE) per swab, a measure of infection intensity, was determined using a Taqman quantitative PCR assay designed for *Bd* quantification (Boyle *et al*., 2004). All samples were run in duplicate; if replicate runs showed inconsistencies in infection status (infected vs. uninfected) or at least one order of magnitude difference in infection intensity, a third replicate was run, and the two most similar replicates were retained.

### Leucocyte counts

After drying, blood smears were fixed in 100% methanol for 5 min, stained with DipQuick (MWI Veterinary Supply, Boise, ID, USA), and examined at ×1000 magnification using a standard light microscope. For each smear, 100 leucocytes were counted and identified as neutrophils (N), lymphocytes (L), eosinophils, monocytes or basophils (Heatley and Johnson, 2009). Numbers of each cell type were calculated as percentages of total leucocytes. The ratio of neutrophils to lymphocytes was calculated as N/L × 100.

### Bacterial killing assay

Bacterial killing ability of *L. yavapaiensis* plasma was tested against *Escherichia coli* (ATCC8739) as described previously (Terrell *et al*., 2013). Briefly, a colony was isolated on a 5% blood agar plate (Fisher Scientific) and used to inoculate a working stock solution. The bacterial concentration of the stock solution was determined by plating serial dilutions (10^−4^, 10^−5^, 10^−6^ and 10^−7^) onto 5% blood agar. For each sample, 5 µl of thawed plasma was combined with 20 µl of bacteria (diluted to 10^6^ colony-forming units ml^−1^) and 75 µl of amphibian phosphate-buffered saline (PBS) in duplicate in a 96-well plate. Plates were shaken and subsequently incubated (21°C, 1 h) to allow bacterial killing to occur. Tryptic soy broth (TSB; 200 µl) was then added to each well, and samples were mixed manually by pipetting to minimize contamination risk, given the relatively large reaction volumes. Plates were incubated for 12 h at 30°C to allow bacterial growth to occur. Given that red blood cell contamination can increase absorbance, a plasma blank (i.e. plasma and PBS only) was included for each sample. Negative (TSB and PBS only) and positive (plasma-free) controls were included in each plate in triplicate. Absorbance was read at 405 nm immediately after incubation. Bacterial killing ability (BKA) was calculated as follows:

$$\frac{\left(A_{\mathrm{sample}}-A_{\mathrm{plasma}\;\mathrm{blank}}\right)}{A_{\mathrm{positive}\;\mathrm{control}}},$$

where *A* is the absorbance at 405 nm.

### Statistical analyses

Frogs euthanized early (≤15 days) after *Bd* exposure were classified only as *Bd* negative or *Bd* positive for statistical analyses, because adequate time had not passed to determine infection or chytridiomycosis susceptibility. In contrast, *Bd*-infected individuals euthanized on or after day 29 were classified, based on observed phenotypes and prior knowledge of infection and susceptibility dynamics from previous experimental and field-based chytridiomycosis studies in *L. yavapaiensis* (Savage and Zamudio, 2011; Savage *et al*., 2011, 2015), into the following two groups: individuals dying of chytridiomycosis (all signs of chytridiomycosis manifesting at the time of euthanasia); and individuals surviving *Bd* infection (no signs of chytridiomycosis manifesting at any time in the 29–55 days before euthanasia). The average daily growth rate was calculated as the total change in body mass (from week 1 to euthanasia) divided by the number of days between the initial and final measurement. In uninfected frogs, there was no significant effect of euthanasia period (i.e. early vs. late) on growth rates (*t *= 1.87, *P *= 0.095), BKA (*t *= −1.60, *P *= 0.124) or N/L ratios (*t *= −1.35, *P *= 0.194); therefore, these control animals were pooled to increase statistical power. Data were analysed using R statistical software (R Development Core Team, 2008). Quantile–quantile plots were used to verify approximate normality in transformed data. Percentage data (i.e. bacterial killing activity and individual leucocyte counts) were arcsine transformed before analysis. All data were back-transformed for presentation in figures. Comparisons made between two groups (i.e. *Bd* exposed vs. unexposed or early vs. late *Bd* exposure) were performed using unpaired Student’s *t*-tests. Differences among susceptible, tolerant and uninfected groups were evaluated by ANOVA and Tukey’s honest significant difference (HSD) test. Principal components analyses were conducted to visualize relationships among response variables (infection load, body mass, BKA and N/L ratios) and animal groups using the *prcomp* function in the base package of R. Only components with eigenvalues >1 were retained in the final analysis (Kaiser, 1960).

## Results

### Experimental infections

Among 49 *Bd*-exposed frogs, 37 individuals had not developed clinical signs of chytridiomycosis on their scheduled euthanasia date [ranging from 1 to 41 days post-infection (DPI); *n* = 27] or at the end of the experiment (55 DPI; *n* = 10 frogs). Eleven of the remaining 12 infected frogs developed all signs of chytridiomycosis and were euthanized immediately (ranging from 34 to 50 DPI) to collect fresh samples before they died; the 12th frog died before euthanasia could take place (31 DPI), and we were unable to collect any viable samples.

All infected frogs shed visible sections of epidermis three or four times per week from 14 DPI onwards, but only the ‘dying’ category of frogs developed other signs of chytridiomycosis. None of the uninfected control frogs (*n* = 28) shed visible sections of epidermis or developed any other signs of chytridiomycosis. Hereafter, *Bd* exposed frogs (*n* = 49) are referred to as ‘early infection’ if they were euthanized ≤15 DPI (before susceptibility could manifest; *n* = 18), ‘surviving’ if they did not develop disease signs by their scheduled ≥29 DPI euthanasia date (*n* = 19) or ‘dying’ if they developed all chytridiomycosis disease signs before euthanasia (*n* = 12). Given that the 12 dying frogs manifested all signs of chytridiomycosis over a 21 day period (ranging from 29 to 50 DPI; median = 40 DPI), some of the ‘surviving’ group of frogs that were euthanized between 29 and 55 DPI may have been *Bd* susceptible but not yet manifesting signs. Thus, the surviving group is likely to have included some susceptible frogs.

**Figure 1:**
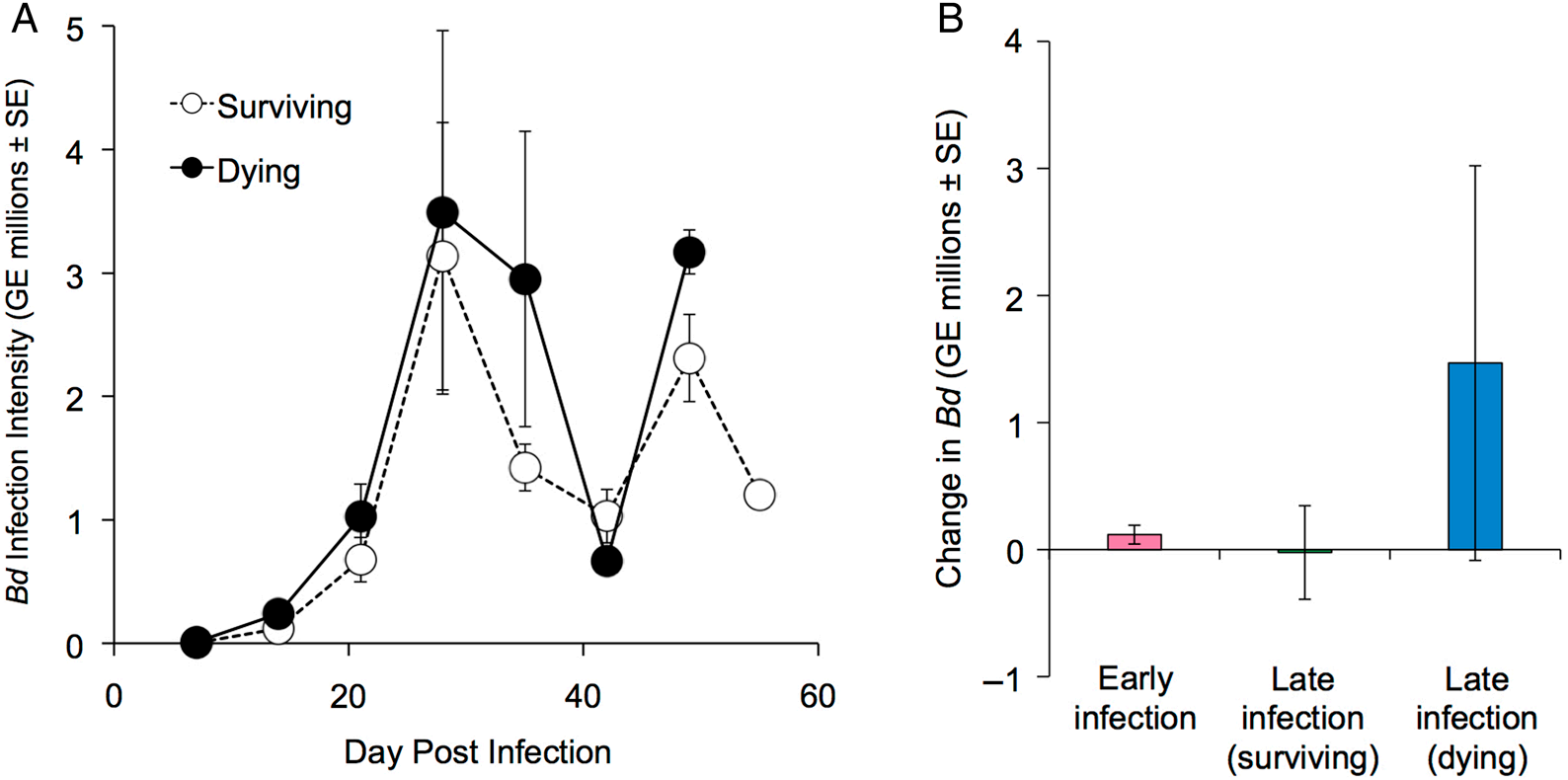
(**A**) Mean *Batrachochytrium dendrobatidis* (*Bd*) infection intensity [measured as genome equivalents (GE)] in dying (filled circles, *n* = 12) and surviving frogs (open circles, *n* = 37) measured weekly from 7 to 55 days post-infection (DPI). (**B**) Change in *Bd* infection intensity (value at time of euthanasia minus value at the most recent weekly sampling) for *Bd*-exposed frogs euthanized 1–15 DPI (pink, *n* = 18) and *Bd*-infected frogs surviving 29–55 DPI with (blue, *n* = 12) or without (green, *n* = 19) signs of chytridiomycosis. All values are shown ±SEM.

Infection intensity rose steadily over time, remained elevated throughout the remainder of the experiment, and was not significantly different between surviving and dying frogs (Fig. 1A; Supplementary Appendix 1 and Supplementary Fig. 1), consistent with field studies of *Bd* in natural *L. yavapaiensis* populations (Savage *et al*., 2011, 2015). From day 29 until 55 DPI, dying frogs showed a non-significant trend towards higher infection loads compared with surviving frogs, with the exception of 42 DPI when all infected frogs had a significant reduction in infection intensity (*q *≥ 5.20, *P *≤ 0.02; Fig. 1A). Based on our qualitative observations that skin shedding was most severe in the week before 42 DPI, we hypothesize that this reduction in zoospore load resulted from a high turnover of epidermis. However, we did not collect quantifiable information on skin shedding. Infection intensity did not change significantly during the week before euthanasia for any infected group (Fig. 1B), consistent with previous observations that *Bd* zoospore load does not reliably predict host mortality in *L. yavapaiensis* (Savage *et al.*, 2011, 2015).

### Leucocyte counts

**Figure 2:**
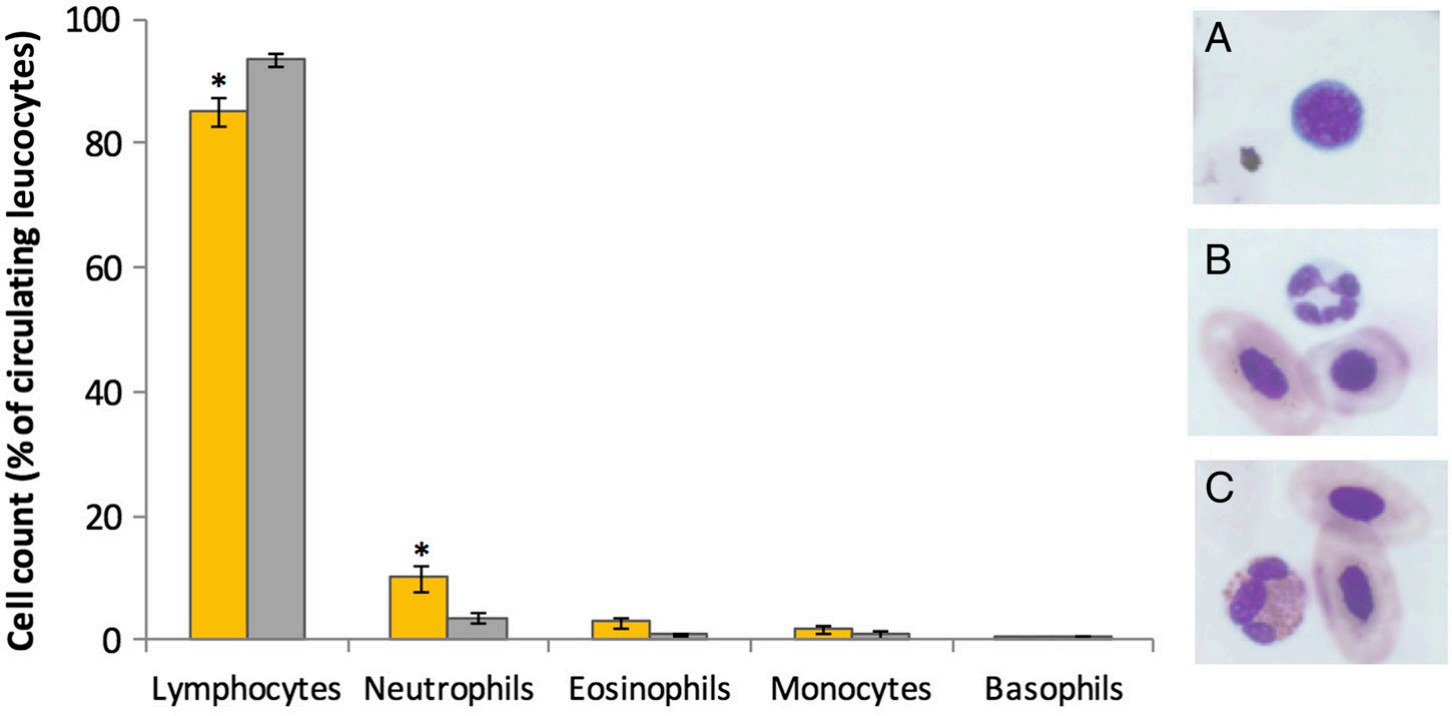
Leucocyte profiles of frogs infected with *Batrachochytrium dendrobatidis* (*Bd*; orange bars, *n* = 26) compared with uninfected control animals (grey bars, *n* = 12) pooled across all time points and survival categories. Representative images of lymphocytes (**A**), neutrophils (**B**) and eosinophils (**C**) in *L. yavapaiensis* are illustrated. Bars with an asterisk (*) differed from control values (*P* < 0.05).

**Figure 3:**
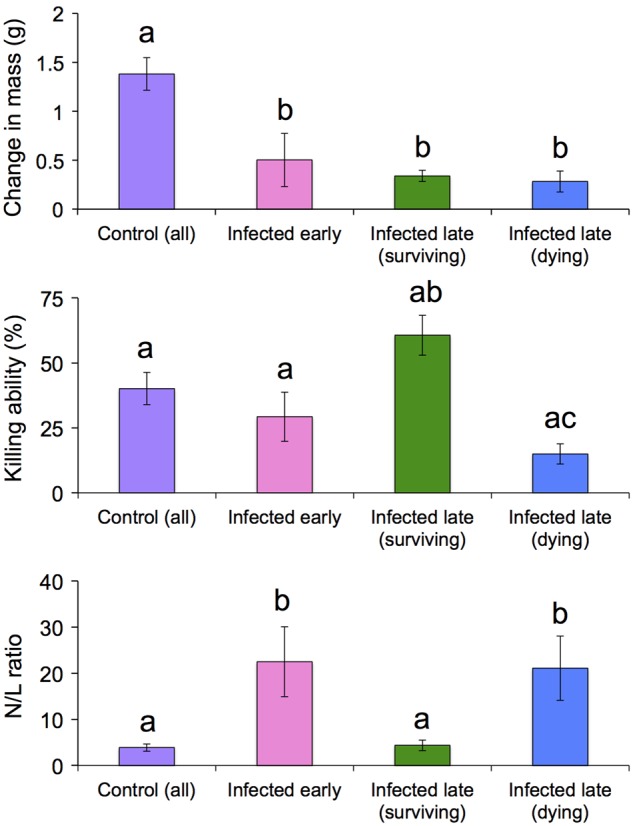
Physiological metrics among frogs exposed to *Batrachochytrium dendrobatidis* (*Bd*) and uninfected control frogs euthanized at early time points [1–15 days post-infection (DPI)] compared with late time points (29–55 DPI). Sample sizes vary among these metrics owing to lack of growth data for week 1 and limited blood volumes for many frogs. Top panel: change in mass in control frogs (*n* = 26), *Bd*-exposed frogs euthanized 1–15 DPI (*n* = 13) and *Bd*-infected frogs surviving 29–55 DPI with (*n* = 10) or without (*n* = 19) signs of chytridiomycosis. Middle panel: bacterial killing ability (BKA) of blood plasma from control frogs (*n* = 26), *Bd*-exposed frogs euthanized early (1–15 DPI, *n* = 18) and *Bd*-infected frogs surviving 29–55 DPI with (*n* = 10) or without (*n* = 17) signs of chytridiomycosis. Bottom panel: ratios of circulating neutrophils to lymphocytes (N/L) in control frogs (*n* = 12), *Bd*-exposed frogs euthanized early (1–15 DPI, *n* = 8) and *Bd*-infected frogs surviving 29–55 DPI with (*n* = 6) or without (*n* = 12) signs of chytridiomycosis. Groups with different letters represent statistically significant differences (*P* < 0.05). All values are shown ±SEM.

Blood samples were obtained in sufficient volume and quality (i.e. without clotting) to permit leucocyte counts in 38 individuals (*n* = 12 control, *n* = 8 early infection, *n* = 12 surviving and *n* = 6 dying). Across all samples (*n* = 38; Supplementary Appendix 1), lymphocytes were the predominant leucocyte in circulation (mean ± SEM, 88.0 ± 1.8%) followed by neutrophils (8.0 ± 1.5%; Fig. 2). Percentages of neutrophils were increased (*t *= −2.47, *P *= 0.018) and percentages of lymphocytes decreased (*t *= 2.85, *P *= 0.007) in infected vs. control animals. Given that changes in both cell types were consistent with a response to stress or infection, we focused subsequent analysis on N/L ratios, a single metric that is commonly used to quantify stress in vertebrates (Davis *et al.*, 2008). These ratios differed (*F *= 6.38, *P *= 0.002) among uninfected, early infection, dying and surviving groups (Fig. 3). Specifically, *post hoc* comparisons revealed that the N/L ratio was increased in early infection *Bd*-positive frogs (*q *= 4.77, *P *= 0.010) and in dying frogs (*q *= 4.02, *P *= 0.036) compared with uninfected control animals. In contrast, surviving frogs demonstrated N/L ratios that were lower than other *Bd*-positive groups (*q* ≥ 3.91, *P* ≤ 0.043) and similar to uninfected control frogs. Other cell types were observed infrequently (eosinophils, 2.2 ± 0.6%; monocytes, 1.5 ± 0.4%; and basophils, 0.3 ± 0.1%), and proportions did not differ among all groups (Fig. 2).

### Average daily growth rate

Given that initial mass was recorded on the first day of the experiment, growth rates were not calculated for animals euthanized in the first 7 days (*n* = 3 control, *n* = 5 *Bd* exposed). Average daily growth rates differed (*F *= 11.26, *P* < 0.0001) among groups. Specifically, growth was reduced (*q* ≥ 5.23, *P *≤ 0.003) in *Bd*-exposed frogs (*n* = 42, including early infection, dying and surviving groups) compared with uninfected control frogs (*n* = 26; Fig. 3). Growth rates were similar among early infection (*n* = 13), dying (*n* = 10) and surviving (*n* = 19) groups.

### Bacterial killing ability

Sufficient volumes of blood were obtained to assay BKA in 72 individuals (*n* = 27 control, *n* = 18 early infection, *n* = 17 surviving and *n* = 10 dying). Animal groups differed (*F = *5.96, *P *= 0.001) with respect to BKA. *Post hoc* analysis revealed that BKA of surviving frogs was increased compared with dying frogs (*q *≥ 4.57, *P *≤ 0.01). However, neither group differed from uninfected control animals or frogs euthanized within 15 days of *Bd* exposure (Fig. 3), consistent with a sorting of surviving and dying frogs associated with initial BKA.

### Principal components analysis

**Figure 4:**
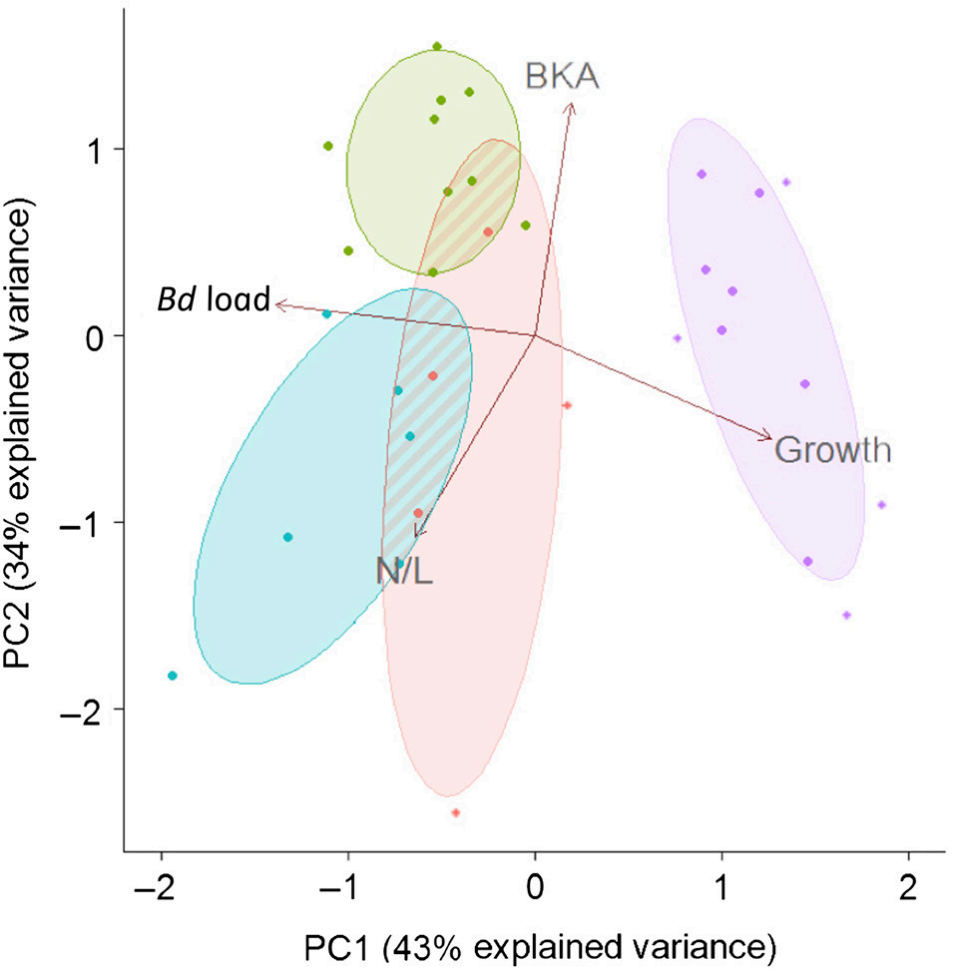
Principal components analysis (PCA) illustrating relationships among *Batrachochytrium dendrobatidis* (*Bd*) load, bacterial killing ability (BKA), growth rates and neutrophil/lymphocyte (N/L) ratios in uninfected frogs (*n* = 11, purple), *Bd*-infected individuals that did not develop chytridiomycosis (*n* = 10, green), *Bd*-infected individuals with chytridiomycosis (*n* = 6, blue) or *Bd*-exposed individuals euthanized during early infection, before disease signs occurred (*n* = 5, pink). Corresponding ellipses represent 68% confidence intervals for each group. Individuals with missing data for any of the above parameters were excluded from the PCA by necessity, resulting in smaller sample sizes.

Frogs with missing data were excluded from PCA, resulting in smaller sample sizes for uninfected (*n*= 11), early infection (*n* = 5), surviving (*n* = 10) and dying (*n* = 6) frogs. Missing data were the result of limited blood volumes, which precluded N/L or BKA analysis for some frogs. Principal components 1 and 2 collectively accounted for 77% of the observed variation in the data set (Fig. 4). Visualization of these components revealed that dying frogs, survivors and control animals represented three distinct physiological groups. Frogs surviving infection were associated with high BKA, low N/L ratios, intermediate body mass gain and high zoospore loads. In contrast, frogs dying of chytridiomycosis exhibited low BKA, high N/L ratios, low body mass gain and high zoospore loads. Control frogs were associated with high body mass gain, but substantial variation in immune function.

## Discussion

Hallmarks of effective immunity against a spectrum of pathogens are well described among endothermic vertebrates. In contrast, amphibian immunology is a relatively nascent field, with little known about protective immune response characteristics outside of the model system *Xenopus*/*Silurana* (Du Pasquier *et al*., 1989). In particular, few studies have examined anuran haematological responses to *Bd* infection and chytridiomycosis (Gervasi *et al*., 2013). In the present study, we found that the complement pathway of the innate immune system, as inferred by plasma BKA against *E. coli*, is a significant predictor of *Bd* susceptibility in this host species (Figs 3 and 4). However, the importance of complement for *Bd* susceptibility in other species and the precise role of complement activity in limiting *Bd* damage to the host remain to be tested (Figs 3 and 4). An equally important finding is that uninfected individuals showed a broad range of BKA, suggesting that baseline levels of circulating complement proteins vary widely among individuals and might be a useful metric for predicting *Bd* susceptibility in individuals or populations. Our interpretation of these findings is complicated, however, by the fact that complement proteins can be produced locally in the epidermis (i.e. the *Bd* infection site) or imported from the bloodstream (Dovezenski *et al*., 1992). Given that we measured only BKA of blood-based complement, we do not know how these values correspond to those at the site of infection. Thus, it is difficult to know whether the observed relationship between blood BKA and *Bd* tolerance is correlative or causative.

When used with fresh whole blood, the BKA is a broad-spectrum challenge assay testing both humoral and cell-mediated killing mechanisms present in circulating blood, including antimicrobial peptides, lysozyme, complement, monocytes, neutrophils and natural killer cells (Janeway *et al*., 2001). In contrast, when plasma or serum is used the white bloods cells have been removed, and therefore, only humoral immune mechanisms are assessed. Given that we used the Gram-negative bacteria *E. coli* as our pathogen, and lysozyme is primarily effective against Gram-positive bacteria, the BKA in our study was likely to be an assessment of complement activity. Our study provides an interesting comparison to the whole-blood BKA results in the only other study investigating *Bd*-induced immune parameters in a North American ranid frog, *R. cascadae* (Gervasi *et al*., 2013). Although we found no significant increase in plasma BKA in infected surviving frogs, infected surviving *R. cascadae* individuals had significantly stronger blood bactericidal activity than control frogs (Gervasi *et al*., 2013). This difference suggests that effective immune responses against *Bd* may involve humoral mechanisms other than complement, that there are species- or strain-specific differences in immune response to *Bd*, that environmental differences between our studies dictate frog immune function, or some combination thereof.

Compared with BKA values, baseline levels of blood leucocytes were more consistent. Among *Bd*-infected frogs, we observed an increase in the ratios of circulating neutrophils to lymphocytes (N/L), consistent with an infection and/or a stress response. Interestingly, this response was not sustained among individuals that survived infection (Figs 3 and 4). Thus, blood N/L ratios may also be an important hallmark of severe chytridiomycosis, with persistently high values indicating susceptibility and a return to baseline values predicting survival. Elevated N/L ratios may result from the loss of lymphocytes in circulation, as these cells migrate from the blood into the tissues. Given the evidence for *Bd*-induced lymphocyte apoptosis (Fites *et al*., 2013), this cellular response may be ineffective in frogs with chytridiomycosis. Whether resistant frogs are able to protect their lymphocytes from destruction by *Bd* is an intriguing question for future research. Elevated N/L ratios may also arise from a greater input of neutrophils into the blood from the bone marrow, where a large reserve of mature neutrophils is maintained (Furze and Rankin, 2008). Neutrophil mobilization is a rapid and crucial component of the inflammatory response. However, a sustained inflammatory response can be highly damaging to the host (Sears *et al*., 2011) and may directly contribute to death in *Bd*-susceptible amphibians. Consistent with this idea, expression of pro-inflammatory genes is elevated during *Bd* infection in four frog species, and expression levels correlate positively with susceptibility (Ellison *et al*., 2015). Likewise, infected *R. cascadae* individuals exhibit elevated N/L ratios but no significant change in lymphocyte counts (Gervasi *et al*., 2013), implying that the ratio change was attributable to an increase in pro-inflammatory neutrophils. Given the small volumes of blood available, we did not perform total (i.e. complete) blood counts in *L. yavapaiensis* and therefore cannot resolve whether changes in N/L ratios were driven by neutrophil or lymphocyte counts (or both). Here, we can only conclude that cell-mediated processes play some role in determining chytridiomycosis outcomes, meriting further investigation across a range of host amphibians.

We found that *Bd* infection intensity significantly reduced growth rate, supporting the well-established trade-off between vertebrate immune responses and fitness that occurs as the host redistributes nutritional and energy resources from anabolic to disease-fighting processes (Lochmiller and Deerenberg, 2000). Several non-exclusive processes may explain this trade-off in our study. Immune cells consume substantial amounts of energy (Newsholme and Newsholme, 1989), thus fitness trade-offs are most pronounced for the acquired (and primarily cell-mediated) immune system (Fearon and Locksley, 1996). However, the extent to which acquired immune responses are mobilized in response to *Bd* infection is unknown. One possibility is that acquired immune responses play a larger role in amphibian responses to infection than previously appreciated and that the negative relationship between infection load and body mass was driven by proliferation of immune cells. Alternately, *Bd* infection might primarily activate the innate immune system, but these responses might be more energetically expensive than previously appreciated. Furthermore, infected animals might reduce food intake (Kyriazakis *et al*., 1998) or absorb nutrients less efficiently, either of which might contribute to declines in growth or body condition (Lochmiller and Deerenberg, 2000). In the present study, we monitored food intake qualitatively and observed cessation of eating among dying frogs, which was one of the criteria for diagnosing chytridiomycosis. However, infected surviving frogs continued to eat normally throughout the experiment, suggesting that their lower gain in mass compared with control animals was attributable to energetic costs of infection, not a reduction in food intake. Finally, the skin shedding we observed among all infected frogs might have contributed substantially to the observed energetic costs of infection. Other studies of amphibian chytridiomycosis have found correlations between decreased mass and increased skin shedding among infected animals (Murphy *et al*., 2011; Peterson *et al*., 2013), and the rate of skin shedding is positively correlated with *Bd* infection intensity in the tree frog *L. caerulea* (Ohmer *et al*., 2015). Discriminating among which immunological or other physiological parameters drive the metabolic changes associated with chytridiomycosis is therefore an important avenue of continued research.

Given that diseased animals are unlikely to survive in natural habitats, it has been suggested that the rapid, innate immune response is the most important determinant of survival and fitness (Lochmiller and Deerenberg, 2000). This is considered particularly true for short-lived species facing a high risk of predation (Lee, 2006). Here, we found that some frogs were able to maintain fair body condition with no signs of disease, despite high *Bd* loads, for at least 55 days after infection. This finding suggests that, even in the case of a highly infectious pathogen and a relatively short-lived host, certain individuals might survive infection long enough for adaptive immune responses to be effective. However, precise characterization of innate vs. adaptive immune activation in amphibians infected with *Bd* is needed before we can draw broad conclusions about the relative roles of different immune pathways in combating chytridiomycosis. Interestingly, some natural populations of *L. yavapaiensis* maintain similarly high *Bd* loads [millions of genome equivalents (GE)] with no indication of morbidity or mortality from chytridiomycosis, whereas other populations exhibit chytridiomycosis die-offs at significantly lower infection intensities, and climatic factors predict these differences (Savage *et al*., 2015). This pattern holds across a range of amphibian species and geographical regions; temperature largely explains the variation in *Bd* infection intensity among individuals in a number of recent field- and laboratory-based studies (Murray *et al.*, 2013; Fernández-Beaskoetxea *et al.*, 2015; Tinsley *et al.*, 2015).

Experimental *Bd* infections also find that amphibians can survive and recover from signs of chytridiomycosis, but maintain *Bd* infection indefinitely (Carver *et al*., 2010). Likewise, our experimental study provides further evidence that infection intensity is not a straightforward predictor of chytridioymycosis susceptibility and suggests that *Bd* tolerance (i.e. limiting pathogen damage rather than pathogen burden) might be an important survival mechanism.

Our study has important implications for understanding the functional immune parameters that influence chytridiomycosis outcomes after *Bd* exposure. We demonstrated that *Bd* infection significantly altered the distribution of circulating host leucocyte types and that this effect was temporary in individuals that could survive and tolerate *Bd* infection, but persisted in susceptible frogs. Although positively associated with *Bd* survival, BKA in uninfected frogs varied widely and did not differ between tolerant or susceptible individuals. We therefore suggest that innate immune function measured by BKA is an important predictor of future susceptibility to chytridiomycosis if exposed to *Bd*. Elevated N/L ratio is also a key biomarker for *Bd* susceptibility among infected animals, and differentiating the relative contribution of increased neutrophils vs. decreased lymphocytes in driving this pattern might be a crucial next step in understanding immune responses against *Bd*. Our study contributes to the growing body of evidence that host immunity is critical for surviving *Bd* infection (Carey *et al*., 1999; Savage and Zamudio, 2011; McMahon *et al*., 2014) and suggests that variation observed across the amphibian world in host tolerance or resistance to *Bd* arises from variation in host immune competence. Here, we find preliminary evidence to suggest that complement-based BKA promotes *Bd* tolerance and enables some individuals to survive despite high pathogen burdens. Whether such tolerance is attributable to genetics, environmental conditions or gene-by-environment interactions remains an unanswered question. Using functional genomic approaches to understand the precise conditions, time points and molecular pathways that can promote effective host immunity and reduce *Bd* pathogenicity is a crucial next step in mitigating amphibian declines.

## Supplementary material

Supplementary material is available at *Conservation Physiology* online.

## Funding

This work was supported by a Smithsonian Institution Competitive Grants Program for Science grant to A.E.S., B.G. and R.C.F., the Smithsonian’s Center for Conservation and Evolutionary Genetics, a Smithsonian Institution Molecular Evolution Postdoctoral Fellowship to A.E.S., and a Smith Postdoctoral Fellowship to K.A.T.

## Supplementary Material

Supplementary Data

## References

[COW011C1] Bennett MF, Alspaugh JK (1964) Some changes in the blood of frogs following administration of hydrocortisone. Virginia J Sci 15: 76–79.

[COW011C2] Bennett MF, Gaudio CA, Johnson AO, Spisso JH (1972) Changes in the blood of newts, *Notophthalmus viridescens*, following administration of hydrocortisone. J Comp Physiol A 80: 233–237.

[COW011C3] Berger L, Speare R, Daszak P, Greene DE, Cunningham AA, Goggin CL, Slocombe R, Ragan MA, Hyatt AD, McDonald KR et al (1998) Chytridiomycosis causes amphibian mortality associated with population declines in the rain forests of Australia and Central America. Proc Natl Acad Sci USA 95: 9031–9036. 9671799 10.1073/pnas.95.15.9031 PMC21197

[COW011C4] Berger L, Speare R, Kent A (2000) Diagnosis of chytridiomycosis in amphibians by histologic examination. Zoo Print J 15: 184–190.

[COW011C5] Berger L, Speare R, Skerratt LF (2005) Distribution of *Batrachochytrium dendrobatidis* and pathology in the skin of green tree frogs *Litoria caerulea* with severe chytridiomycosis. Dis Aquat Organ 68: 65–70. 16465835 10.3354/dao068065

[COW011C6] Blaustein AR, Gervasi SS, Johnson PT, Hoverman JT, Belden LK, Bradley PW, Xie GY (2012) Ecophysiology meets conservation: understanding the role of disease in amphibian population declines. Philos Trans R Soc B Biol Sci 367: 1688–1707. 10.1098/rstb.2012.0011 PMC3350657 22566676

[COW011C700] Boyle DG, Boyle DB, Olsen V, Morgan JA, Hyatt AD (2004) Rapid quantitative detection of chytridiomycosis (*Batrachochytrium dendrobatidis*) in amphibian samples using real-time Taqman PCR assay. Dis Aquat Organ 60: 141–148. 15460858 10.3354/dao060141

[COW011C7] Bradley G, Rosen P, Sredl M, Jones T, Longcore J (2002) Chytridiomycosis in native Arizona frogs. J Wild Dis 38: 206–212. 10.7589/0090-3558-38.1.206 11838218

[COW011C8] Campbell TW (2015) Peripheral Blood of Amphibians. Wiley-Blackwell, Malden.

[COW011C9] Carey C (1993) Hypothesis concerning the causes of the disappearance of boreal toads from the mountains of Colorado. Conserv Biol 7: 355–362.

[COW011C10] Carey C, Cohen N, Rollins-Smith L (1999) Amphibian declines: an immunological perspective. Dev Comp Immunol 23: 459–472. 10512457 10.1016/s0145-305x(99)00028-2

[COW011C11] Carver S, Bell BD, Waldman B (2010) Does chytridiomycosis disrupt amphibian skin function? Copeia 2010: 487–495.

[COW011C12] Cathers T, Lewbart GA, Correa M, Stevens JB (1997) Serum chemistry and hematology values for anesthetized American bullfrogs (*Rana catesbeiana*). J Zoo Wild Med 28: 171–174. 9279406

[COW011C14] Davis AK, Maney DL, Maerz JC (2008) The use of leukocyte profiles to measure stress in vertebrates: a review for ecologists. Funct Ecol 22: 760–772.

[COW011C15] Davis AK, Keel MK, Ferreira A, Maerz JC (2010) Effects of chytridiomycosis on circulating white blood cell distributions of bullfrog larvae (*Rana catesbeiana*). Comp Clin Pathol 19: 49–55.

[COW011C16] Densmore CL, Green DE (2007) Diseases of amphibians. Inst Lab Anim Res J 48: 235–254. 10.1093/ilar.48.3.235 17592186

[COW011C17] Dovezenski N, Billetta R, Gigli I (1992) Expression and localization of proteins of the complement system in human skin. J Clin Invest 90: 2000–2012. 1385479 10.1172/JCI116080 PMC443264

[COW011C18] Du Pasquier L, Schwager J, Flajnik MF (1989) The immune system of *Xenopus*. Ann Rev Immunol 7: 251–275. 2653371 10.1146/annurev.iy.07.040189.001343

[COW011C19] Ellison AR, Tunstall T, DiRenzo GV, Hughey MC, Rebollar EA, Belden LK, Harris RN, Ibanez R, Lips KR, Zamudio KR (2015) More than skin deep: functional genomic basis for resistance to amphibian chytridiomycosis. Genome Biol Evol 7: 286–298. 10.1093/gbe/evu285 PMC4316636 25539724

[COW011C20] Fearon DT, Locksley RM (1996) The instructive role of innate immunity in the acquired immune response. Science 272: 50–55. 8600536 10.1126/science.272.5258.50

[COW011C21] Fernández-Beaskoetxea S, Carrascal LM, Fernández-Loras A, Fisher MC, Bosch J (2015) Short term minimum water temperatures determine levels of infection by the amphibian chytrid fungus in *Alytes obstetricans* tadpoles. PLoS ONE 10: e0120237. 25793985 10.1371/journal.pone.0120237 PMC4368698

[COW011C22] Fites JS, Ramsey JP, Holden WM, Collier SP, Sutherland DM, Reinert LK, Gayek AS, Dermody TS, Aune TM, Oswald-Richter K et al (2013) The invasive chytrid fungus of amphibians paralyzes lymphocyte responses. Science 342: 366–369. 24136969 10.1126/science.1243316 PMC3956111

[COW011C23] Flajnik MF, Kasahara M (2001) Comparative genomics of the MHC: glimpses into the evolution of the adaptive immune system. Immunity 2001: 351–362. 10.1016/s1074-7613(01)00198-4 11567626

[COW011C24] Forrest MJ, Schlaepfer MA (2011) Nothing a hot bath won’t cure: infection rates of amphibian chytrid fungus correlate negatively with water temperature under natural field settings. PLoS ONE 6: e28444. 22205950 10.1371/journal.pone.0028444 PMC3244395

[COW011C25] Furze RC, Rankin SM (2008) Neutrophil mobilization and clearance in the bone marrow. Immunology 125: 281–288. 19128361 10.1111/j.1365-2567.2008.02950.x PMC2669132

[COW011C26] Gervasi SS, Hunt EG, Lowry M, Blaustein AR (2013) Temporal patterns in immunity, infection load and disease susceptibility: understanding the drivers of host responses in the amphibian-chytrid fungus system. Funct Ecol 28: 569–578.

[COW011C27] Hayman J, Bly J, Levine R, Lobb C (1992) Complement deficiencies in channel catfish (*Ictalurus punctatus*) associated with temperature and seasonal mortality. Fish Shellfish Immunol 2: 183–192.

[COW011C13] Heatley JJ, Johnson M (2009) Clinical technique: amphibian hematology: a practitioner’s guide. J Exot Pet Med 18: 14–19.

[COW011C28] Hedrick MS, Hillman SS, Drewes RC, Withers PC (2013) Lymphatic regulation in nonmammalian vertebrates. J Appl Physiol 115: 297–308. 23640588 10.1152/japplphysiol.00201.2013

[COW011C29] Hyatt AD, Boyle DG, Olsen V, Boyle DB, Berger L, Obendorf D, Dalton A, Kriger K, Heros M, Hines H et al (2007) Diagnostic assays and sampling protocols for the detection of *Batrachochytrium dendrobatidis*. Dis Aquat Organ 73: 175–192. 17330737 10.3354/dao073175

[COW011C30] Jain NC (1993) Essentials of Veterinary Hematology. Blackwell Publishing, Philadelphia.

[COW011C31] Janeway CAJ, Travers P, Walport M, Shlomchik MJ (2001) Immunobiology. Garland Science, New York.

[COW011C32] Jiang C, Zhang J, Yao J, Liu S, Li Y, Song L, Li C, Wang X, Liu Z (2015) Complement regulatory protein genes in channel catfish and their involvement in disease defense response. Dev Comp Immunol 53: 33–41. 26111998 10.1016/j.dci.2015.06.002

[COW011C33] Kaiser HF (1960) The application of electronic computers to factor analysis. Ed Psych Measurement 20: 141–151.

[COW011C35] Koppenheffer TL (1987) Serum complement-systems of ectothermic vertebrates. Dev Comp Immunol 11: 279–286. 3305102 10.1016/0145-305x(87)90072-3

[COW011C36] Kyriazakis I, Tolkamp BJ, Hutchings MR (1998) Towards a functional explanation for the occurrence of anorexia during parasitic infections. Anim Behav 56: 265–274. 9787017 10.1006/anbe.1998.0761

[COW011C37] Lee KA (2006) Linking immune defenses and life history at the levels of the individual and the species. Int Comp Biol 46: 1000–1015. 10.1093/icb/icl049 21672803

[COW011C38] Lochmiller RL, Deerenberg C (2000) Tradeoffs in evolutionary immunology: just what is the cost of immunity? Oikos 88: 87–98.

[COW011C39] McMahon TA, Sears BF, Venesky MD, Bessler SM, Brown JM, Deutsch K, Halstead NT, Lentz G, Tenouri N, Young S et al (2014) Amphibians acquire resistance to live and dead fungus overcoming fungal immunosuppression. Nature 511: 224–227. 25008531 10.1038/nature13491 PMC4464781

[COW011C41] Maniero G, Carey C (1997) Changes in selected aspects of immune function in the leopard frog, *Rana pipiens*, associated with exposure to cold. Comp Biochem Physiol B Biochem Mol Biol 167: 256–263. 10.1007/s003600050072 9203367

[COW011C42] Merchant M, Roche C, Elsey RM, Prudhomme J (2003) Antibacterial properties of serum from the American alligator (*Alligator mississippiensis*). Comp Biochem Physiol B Biochem Mol Biol 136: 505–513. 14602158 10.1016/s1096-4959(03)00256-2

[COW011C45] Murphy PJ, St-Hilaire S, Corn PS (2011) Temperature, hydric environment, and prior pathogen exposure alter the experimental severity of chytridiomycosis in boreal toads. Dis Aquat Organ 95: 31–42. 21797033 10.3354/dao02336

[COW011C46] Murray KA, Skerratt LF, Garland S, Kriticos D, McCallum H (2013) Whether the weather drives patterns of endemic amphibian chytridiomycosis: a pathogen proliferation approach. PLoS ONE 8: e61061. 23613783 10.1371/journal.pone.0061061 PMC3629077

[COW011C47] Newsholme P, Newsholme EA (1989) Rates of utilization of glucose, glutamine and oleate and formation of end-products by mouse peritoneal macrophages in culture. Biochem J 261: 211–218. 2775207 10.1042/bj2610211 PMC1138802

[COW011C48] Nielsen CH, Leslie RGQ (2002) Complement’s participation in acquired immunity. J Leukocyte Biol 72: 249–261. 12149415

[COW011C49] Nonaka M, Kimura A (2006) Genomic view of the evolution of the complement system. Immunogenetics 58: 701–713. 16896831 10.1007/s00251-006-0142-1 PMC2480602

[COW011C50] Ohmer ME, Cramp RL, White CR, Franklin CE (2015) Skin sloughing rate increases with chytrid fungus infection load in a susceptible amphibian. Funct Ecol 29: 674–682.

[COW011C51] Ohta Y, Goetz W, Hossain MZ, Nonaka M, Flajnik MF (2006) Ancestral organization of the MHC revealed in the amphibian *Xenopus*. J Immunol 176: 3674–3685. 16517736 10.4049/jimmunol.176.6.3674

[COW011C52] Pessier AP, Nichols DK, Longcore JE, Fuller MS (1999) Cutaneous chytridiomycosis in Poison Dart Frogs (*Dendrobates* spp.) and White’s Tree Frogs (*Litoria caerulea*). J Vet Diagnost Invest 11: 194–199. 10.1177/104063879901100219 10098698

[COW011C53] Peterson JD, Steffen JE, Reinert LK, Cobine PA, Appel A, Rollins-Smith L, Mendonça MT (2013) Host stress response is important for the pathogenesis of the deadly amphibian disease, chytridiomycosis, in *Litoria caerulea*. PLoS ONE 8: e62146. 23630628 10.1371/journal.pone.0062146 PMC3632538

[COW011C54] Ray TL, Wuepper KD (1978) Experimental cutaneous candidiasis in rodents: II. Role of the stratum corneum barrier and serum complement as a mediator of a protective inflammatory response. Archiv Dermatol 114: 539–543. 10.1001/archderm.114.4.539 646364

[COW011C680] R Development Core Team (2008) R: A Language and Environment for Statistical Computing. R Foundation for Statistical Computing, Vienna, Austria.

[COW011C55] Ribas L, Li MS, Doddington BJ, Robert J, Seidel JA, Kroll JS, Zimmerman LB, Grassly NC, Garner TW, Fisher MC (2009) Expression profiling the temperature-dependent amphibian response to infection by *Batrachochytrium dendrobatidis*. PLoS ONE 4: e8408. 20027316 10.1371/journal.pone.0008408 PMC2794374

[COW011C56] Robert J, Ohta Y (2009) Comparative and developmental study of the immune system in *Xenopus*. Develop Dynam 238: 1249–1270. 10.1002/dvdy.21891 PMC2892269 19253402

[COW011C57] Rosenblum EB, Poorten TJ, Settles M, Murdoch GK (2012) Only skin deep: shared genetic response to the deadly chytrid fungus in susceptible frog species. Mol Ecol 21: 3110–3120. 22332717 10.1111/j.1365-294X.2012.05481.x

[COW011C58] Savage AE, Zamudio KR (2011) MHC genotypes associate with resistance to a frog-killing fungus. Proc Natl Acad Sci USA 108: 16705–16710. 21949385 10.1073/pnas.1106893108 PMC3189034

[COW011C59] Savage AE, Sredl MJ, Zamudio KR (2011) Disease dynamics vary spatially and temporally in a North American amphibian. Biol Conserv 144: 1910–1915.

[COW011C60] Savage AE, Becker CG, Zamudio KR (2015) Linking genetic and environmental factors in amphibian disease risk. Evol Appl 8: 560–572. 26136822 10.1111/eva.12264 PMC4479512

[COW011C61] Schlaepfer MA, Sredl MJ, Rosen PC, Ryan MJ (2007) High prevalence of *Batrachochytrium dendrobatidis* in wild populations of lowland leopard frogs *Rana yavapaiensis* in Arizona. EcoHealth 4: 421–427.

[COW011C62] Sears BF, Rohr JR, Allen JE, Martin LB (2011) The economy of inflammation: when is less more? Trends Parasitol 27: 382–387. 21680246 10.1016/j.pt.2011.05.004

[COW011C64] Sparkmann AM, Palacio MG (2009) A test of life-history theories of immune defence in two ecotypes of the garter snake, *Thamnophis elegans*. J Anim Ecol 78: 1242–1248. 19622081 10.1111/j.1365-2656.2009.01587.x

[COW011C65] Sredl MJ, Jennings RD (2005) *Rana chiricahuensis*: Platz and Mecham, 1979, Chiricahua leopard frogs. In Lanoo MJ, eds, Amphibian Declines: the Conservation Status of United States Amphibians. University of California Press, Berkeley, pp. 546–549.

[COW011C66] Sunyer JO, Tort L, Lambris JD (1997) Diversity of the third form of complement, C3, in fish: functional characterization of five forms of C3 in the diploid fish *Sparus aurata*. Biochem J 326: 877–881. 9307040 10.1042/bj3260877 PMC1218745

[COW011C67] Sunyer JO, Zarkadis IK, Lambris JD (1998) Complement diversity: a mechanism for generating immune diversity? Immunol Today 19: 519–523. 9818547 10.1016/s0167-5699(98)01341-3

[COW011C68] Tagami H (1992) The role of complement-derived mediators in inflammatory skin diseases. Arch Dermatol Res 284: S2–S9. 1285651 10.1007/BF00638232

[COW011C69] Terrell KA, Quintero RP, Murray S, Kleopfer JD, Murphy JB, Evans MJ, Nissen BD, Gratwicke B (2013) Cryptic impacts of temperature variability on amphibian immune function. J Exp Biol 216: 4204–4211. 23948472 10.1242/jeb.089896

[COW011C70] Tinsley RC, Coxhead PG, Stott LC, Tinsley MC, Piccinni MZ, Guille MJ (2015) Chytrid fungus infections in laboratory and introduced *Xenopus laevis* populations: assessing the risks for UK native amphibians. Biol Conserv 184: 380–388. 25843959 10.1016/j.biocon.2015.01.034 PMC4380136

[COW011C72] Turner RJ (1988) Amphibians. In Rawley AF, Ratcliffe NA, eds, Vertebrate Blood Cells. Cambridge University Press, Cambridge, pp 129–209.

[COW011C73] Venesky MD, Wilcoxen TE, Rensel MA, Rollins-Smith L, Kerby JL, Parris MJ (2012) Dietary protein restriction impairs growth, immunity, and disease resistance in southern leopard frog tadpoles. Oecologia 169: 23–31. 22038058 10.1007/s00442-011-2171-1

[COW011C74] Woodhams DC, Ardipradja K, Alford RA, Marantelli G, Reinert LK, Rollins-Smith LA (2007) Resistance to chytridiomycosis varies among amphibian species and is correlated with skin peptide defenses. Anim Conserv 10: 409–417.

[COW011C76] Zhao F, Yan C, Wang X, Yang Y, Wang G, Lee W, Zhang Y (2014) Comprehensive transcriptome profiling and functional analysis of the frog (*Bombina maxima*) immune system. DNA Res 21: dst035. 10.1093/dnares/dst035 PMC3925390 23942912

[COW011C77] Zhu Y, Thangamani S, How B, Ding LD (2005) The ancient origin of the complement system. EMBO J 24: 382–394. 15616573 10.1038/sj.emboj.7600533 PMC545819

[COW011C78] Zimmerman LM, Vogel LA, Bowden RM (2010a) Understanding the vertebrate immune system: insights from the reptilian perspective. J Exp Biol 213: 661–671. 20154181 10.1242/jeb.038315

[COW011C79] Zimmerman LM, Paitz RT, Vogel LA, Bowden RM (2010b) Variation in the seasonal patterns of innate and adaptive immunity in the red-eared slider (*Trachemys scripta*). J Exp Biol 213: 1477–1483. 20400632 10.1242/jeb.037770

