# Supplementary material for: Reduced immune function predicts disease susceptibility in frogs infected with a deadly fungal pathogen
Source: Conserv Physiol. 2016 Apr 15;4(1):cow011. doi: 10.1093/conphys/cow011 (PMC4834730; doi:10.1093/conphys/cow011)
Supplement: Supplementary Data [file cow011supp.zip › cow011supp_fig1.pdf]

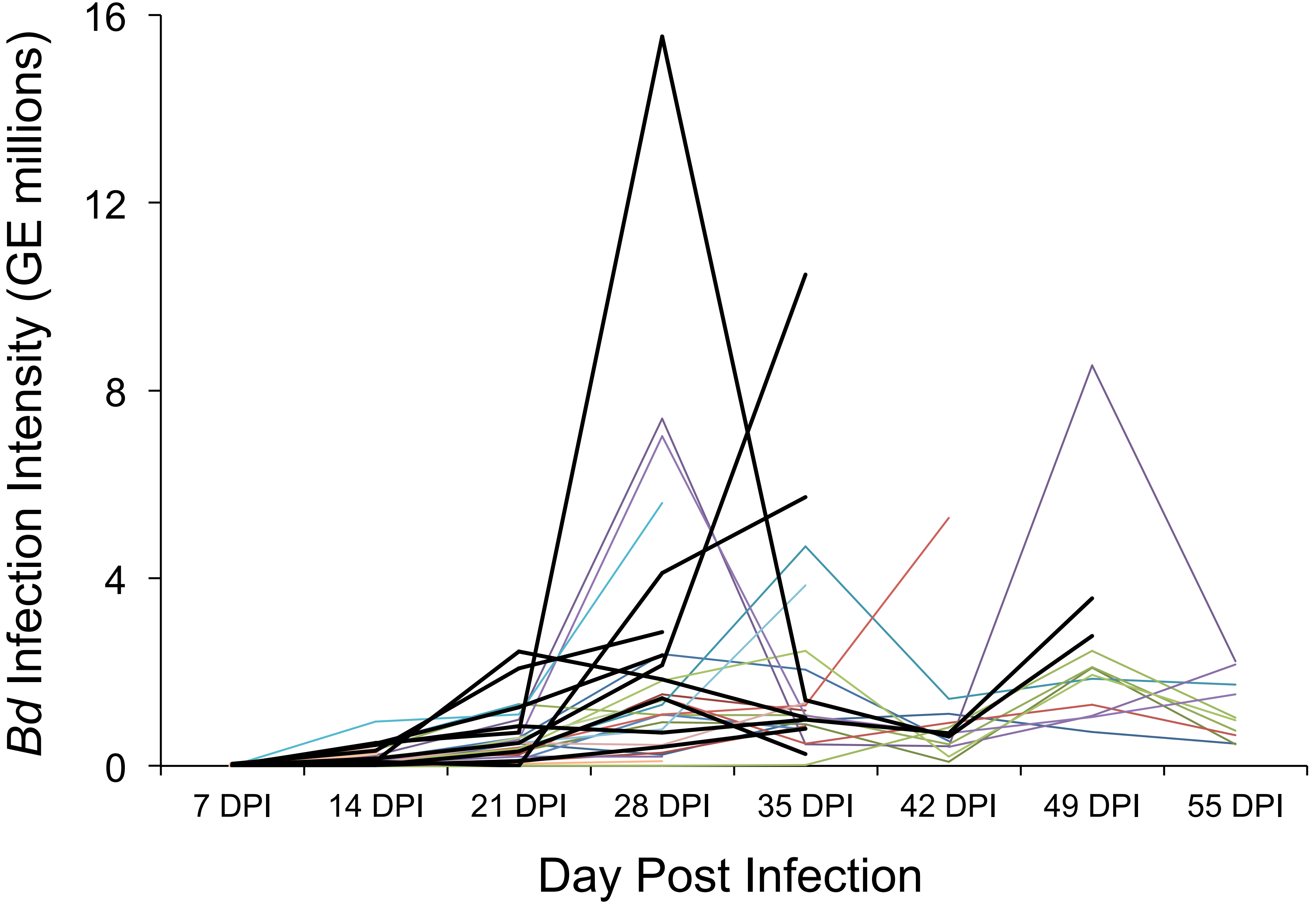

Supplementary Figure 1. Individual *Bd* infection intensity over time for all *Bd* infected frogs. Individuals shown in black represent frogs that were dying with signs of chytridiomycosis at the time of euthanasia.
